# Whey protein fibril-rutin nano-complex: a dual-action strategy to boost antioxidant capacity and mitigate hazardous thermal reactions in roasted beef

**DOI:** 10.1016/j.fochx.2025.103417

**Published:** 2025-12-16

**Authors:** Zening Zhang, Qiaochun Chen, Muhammad Aslam Khan, Baoping Shi, Huaixu Wang, Ka-Wing Cheng

**Affiliations:** a Department of Food Science and Engineering, College of Chemistry and Environmental Engineering, Shenzhen University, Shenzhen 518060, China; b Shenzhen Key Laboratory of Marine Microbiome Engineering, Institute for Advanced Study, Shenzhen University, Shenzhen 518060, China; c Institute for Innovative Development of Food Industry, Shenzhen University, Shenzhen 518060, China; d Institute for Advanced Study, Shenzhen University, Shenzhen 518060, China

**Keywords:** Protein nanofibers, Rutin, Antioxidant activity, Physicochemical property, Heterocyclic amine, Advanced glycation end product, Beef patties

## Abstract

Combined use of whey protein isolate (WPI) and polyphenols have been reported to enhance the quality of thermally-processed food products. WPI fibrils (WPIF) represent a promising WPI derivative with enhanced properties, such as polyphenol-binding capacity and stability. This study investigated the structural and antioxidant characteristics of WPI, WPIF, their rutin complexes, and their effects on hazardous thermal reactions in roasted beef patties. WPIF exhibited significantly higher activity in antioxidant assays than WPI. Rutin encapsulation in WPI and WPIF formed stable complexes WR-E and WFR-E, which effectively attenuated its thermal degradation. These complexes, especially WFR-E, showed significantly enhanced antioxidant capacity than their individual constituents and non-encapsulated mixtures. Moreover, WFR-E demonstrated significantly better inhibition of heterocyclic amines (HAs) and advanced glycation end products (AGEs) formation than the other candidates. These findings suggest the potential of WFR-E as a superior, natural food additive for improving the safety and quality of thermally-processed meat products.

## Highlights

• WPIF exhibited significantly higher antioxidant activity than native WPI.
• Encapsulation of rutin in WPI and WPIF greatly reduced its thermal degradation rate.
• Rutin-encapsulated WPIF (WFR-E) had better antioxidant capacity than other complexes.
• WFR-E effectively reduced TBARS, HAs, and AGEs contents in roasted beef patties.

## Graphical abstract

**Figure:**
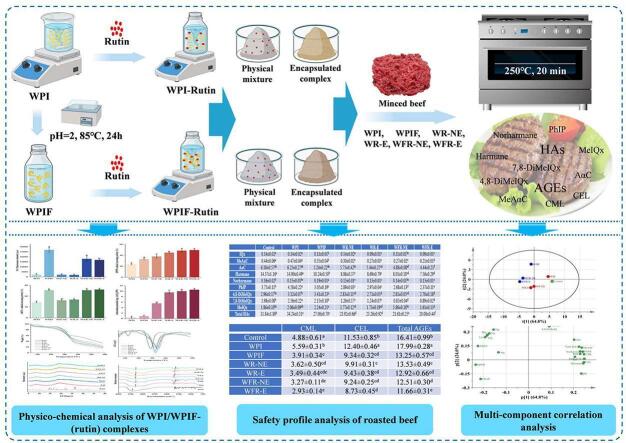

## 1 Introduction

Roasted meat products are widely favored by consumers mainly because of their desirable sensory properties. However, the vigorous thermal conditions can often give rise to hazardous compounds such as heterocyclic amines (HAs), advanced glycation end products (AGEs), lipid and protein oxidation products (Zhang, Chen, et al., 2024). Long-term intake of HAs has been associated with an increased risk of gastrointestinal cancers such as gastric and colorectal cancer (Deng, Chen, et al., 2022). Similarly, AGEs represent a class of chemically stable and structurally complex Maillard reaction products, with N^ε^-(carboxymethyl)lysine (CML) and N^ε^-(carboxyethyl)lysine (CEL) being recognized as representative markers (Zhang, Wang, et al., 2024). Accumulating studies have tended to implicate them in the development and/or progression of a number of chronic diseases, such as diabetes, cardiovascular disease, and atherosclerosis (Uribarri et al., 2015). Given that these harmful byproducts are often concurrently generated during heat treatments of protein-based foods, strategies to simultaneously mitigate their formation would be of significant importance.

Currently, the use of synthetic antioxidants may pose safety concerns, while the application of spices, especially those with strong distinctive aroma and/or color profiles, may negatively impact the sensory quality of the final product (Linghu et al., 2017; Zeng et al., 2014). These adverse factors limit their widespread use in the food industry. Many studies have demonstrated the promising potential of phenolic compounds for inhibiting the formation of harmful substances in thermally processed food. Meanwhile, it has also been reported that the effectiveness of some candidates may be significantly attenuated due to their poor water solubility (Nguyen et al., 2013; Zhang et al., 2023; Zhang et al., 2025). For instance, rutin, a hydrophobic flavonoid found in many fruits and vegetables, has been known for its versatile bioactivities, such as antioxidant and anti-inflammation (Zhang et al., 2025). Although rutin has been used to inhibit protein oxidation and HA formation in meat products, its poor water solubility has been an important factor compromising its dispersibility and thus even distribution upon mixing with food products, especially those with high moisture matrices, such as meat (Nguyen et al., 2013; Zhang et al., 2023; Zhang et al., 2025).

A variety of encapsulation systems utilizing food-grade materials, such as proteins, peptides, polysaccharides, and cyclodextrins, have been shown to effectively improve the dispersibility and thermal stability of hydrophobic phytochemicals (Li et al., 2025; Zhang et al., 2022). Among them, proteins have attracted much attention for polyphenol encapsulation due to their nutritional value, low toxicity, and biocompatibility. They have also been attractive candidates in studies aiming to simultaneously enhance the nutritional and functional properties (e.g., emulsification and water-holding capacity) of meat products. In particular, whey protein isolate (WPI) has been increasingly recognized for its high nutritional value, low cost, and versatile functional properties (Khalesi et al., 2021). Besides application in its native form, some studies have also investigated the performance of its fibril form (WPIF), which may form when WPI undergoes partial hydrolysis and conformational rearrangement during prolonged heating at acidic pH (≈2) at elevated temperatures (e.g., 85 °C), ultimately driving its self-assembly into long protein fibrils  (Zhang et al., 2021). Compared with native WPI, WPIF often exhibited significantly better emulsifying, gelling, foaming and even antioxidant properties that were likely resulted from increased exposure of hydrophobic residues and functional groups caused by the acid-heat treatment (Kroes-Nijboer et al., 2012; Mohammadian & Madadlou, 2016). Moreover, protein fibrils including WPIF have been demonstrated to form more stable complexes with the encapsulated bioactive compound(s), and thus offer stronger protective effect and achieve higher encapsulation efficiency. Mohammadian et al. (2019) showed that embedding curcumin in WPI improved its dispersibility in water by approximately 180-fold, and embedding in WPIF resulted in even greater enhancement in its dispersibility by ∼1200-fold. These previous reports suggest that WPI and WPIF may be able to form complexes with rutin to improve its properties, besides the potential nutritional and functional properties of the protein itself. However, whether these postulated enhanced properties of WPI- and WPIF-rutin complexes could be translated into stronger favorable effects on key quality attributes of thermally processed meat products has not been clearly characterized.

The present study therefore aimed to evaluate the potential of WPI and WPIF for improving the dispersibility and stability of rutin for its subsequent application to enhance the quality of thermally processed meat products using beef patty as a representative model. To better understand the underlying mechanism, comprehensive analyses were performed on five experimental groups, including WPI alone, physically mixed rutin in WPI (WR-NE), rutin encapsulated in WPI (WR-E), physically-mixed rutin in WPIF (WFR-NE), and rutin encapsulated in WPIF (WFR-E)). Key parameters assayed included antioxidant capacity, thermal stability, rutin degradation rate, and effect on pH, lipid peroxidation, as well as formation of hazardous compounds (HAs and AGEs) in roasted beef patties. The findings of this work provide a mechanistic foundation for developing novel, natural, and high-performance food-grade additives for improved food quality and safety.

## 2 Material and methods

### 2.1 Chemicals and materials

Beef tenderloin was purchased from Xiaoxiang Supermarket (Shenzhen, China). WPI (BiPRO 9500) was obtained from Agropur Inc. (USA). Rutin (purity ≥97 %) was purchased from Yuanye Bio-Technology Co., Ltd. (Shanghai, China). Hydrochloric acid, sodium hydroxide, 2-thiobarbituric acid (TBA), sodium borohydride, and sodium tetraborate were obtained from China National Pharmaceutical Group Chemical Reagent Co., Ltd. (Shanghai, China). Thioflavin T (ThT), ferric chloride, 2,2-diphenyl-1-picrylhydrazyl (DPPH), 2,2′-azinobis (3-ethylbenzothiazolin-6- sulfonsaeure) diammoniumsalz (ABTS), 2,4,6-tri(2-pyridyl)-*s*-triazine (TPTZ), ethyl acetate, trichloroacetic acid (TCA), sodium acetate, glutaraldehyde, methanol, and ethanol were purchased from Macklin Biochemical Co., Ltd. (Shanghai, China). Amino acid standards were obtained from Waters Corporation (Milford, MA, USA). CML, CEL, d₄-CML, and d₄-CEL were purchased from Anpel Laboratory Technologies Inc. (Shanghai, China). Heterocyclic amines (harmane, norharmane, PhIP, MeIQx, 4,8-DiMeIQx, 7,8-DiMeIQx, AαC, and MeAαC) were purchased from CATO Research Chemicals Inc. (Eugene, OR, USA). LC-MS grade formic acid, acetonitrile, and methanol were purchased from Thermo Fisher Scientific Inc. (Waltham, MA, USA) and Merck KGaA (Darmstadt, Germany). Oasis MCX (60 mg/3 cc) solid-phase extraction cartridges were obtained from Waters Technologies Co., Ltd. (Shanghai, China).

### 2.2 Preparation of whey protein isolate fibrils (WPIF)

The preparation of WPIF was performed following the method of Meng et al. (2024). Briefly, 10 g of WPI powder was dissol*v*ed in 500 mL of deionized water (2 %, *w*/*v*) and stirred at 1000 rpm for 2 h at room temperature. The solution was then stored overnight at 4 °C. The pH was adjusted to 2.0 using 6 M HCl, followed by centrifugation at 10,000 rpm for 15 min at 4 °C using a refrigerated centrifuge (ESCO, TCR-1500-8, Singapore). The supernatant was collected and incubated at 85 °C for 24 h. After heating, the samples were immediately cooled in an ice bath to obtain WPIF solutions. An unheated and unadjusted WPI solution (2 %, w/v) was used as the control.

### 2.3 Preparation of rutin-encapsulated and non-encapsulated WPI and WPIF complexes

The preparation of encapsulated complexes of WPI and WPIF with rutin was conducted according to the method of  Meng et al. (2024). Rutin was first dissolved completely in anhydrous ethanol. The rutin solution was then added dropwise to the WPI and WPIF solutions at a mass ratio (protein: rutin) of 50:1. The mixture was stirred at 500 rpm at 4 °C in the dark for 2 h to obtain WPI-rutin and WPIF-rutin encapsulated complexes. The non-encapsulated mixtures were prepared by mixing WPI or WPIF with rutin in powder form at a mass ratio of 50:1.

### 2.4 Determination of thioflavin T (ThT) fluorescence

The ThT fluorescence intensity of different samples was measured according to the method of Dong et al. (2023) with slight modifications. ThT was dissolved in phosphate buffer (10 mM, pH 7.0, 150 mM NaCl) to obtain a 0.8 mg/mL stock solution. The stock solution was diluted 50-fold to prepare the ThT working solution. A total of 40 μL of each sample was mixed with 4 mL of the ThT working solution, vortexed for 1 min, and the fluorescence intensity was measured using a multimode microplate reader (Agilent Technologies, Santa Clara, CA, USA) at excitation and emission wavelengths of 440 nm and 480 nm, respecti*v*ely. The background fluorescence of the ThT working solution was subtracted to obtain the corrected ThT fluorescence intensity.

### 2.5 SDS-PAGE analysis

For SDS-PAGE analysis, 2 mL of each sample (2 mg/mL) was mixed with an equal volume of 2× Laemmli sample buffer. The mixture was heated at 95 °C for 10 min. A 10 μL aliquot of each denatured sample was loaded onto a Super-PAGETM pre-cast gel (Bis-Tris, 4–20 %). Electrophoresis was carried out at a constant voltage of 120 *V* until the dye front reached the bottom of the gel. The gel was then stained with 0.5 % Coomassie Brilliant Blue G-250 for 2 h and destained using a methanol/acetic acid/water solution (4:1:5, *v*/v/v). For the WPIF samples, low-molecular-weight proteins were further analyzed using the All-in-One Tricine-PAGE Kit under similar electrophoresis conditions. After electrophoresis, the Tricine gels were fixed in isopropanol-based fixative for 30 min and then stained for 15 min using a rapid Coomassie blue stain, followed by rinsing with pure water.

### 2.6 Thermogravimetric (TG) analysis

The thermal stability of WPI, WPIF, and their complexes with rutin was assessed using a TG analyzer (Mettler-Toledo, Zurich, Switzerland). Samples were heated in aluminum crucibles from 20 °C to 600 °C at a rate of 20 °C/min (Meng et al., 2024). Thermogravimetric curves were recorded, and derivative thermogravimetric (dTG) curves were obtained from the first derivative of the TGA curves.

### 2.7 X-ray diffraction (XRD) analysis

The XRD intensity of different samples was measured according to the method of Meng et al. (2024) with slight modifications. X-ray diffraction patterns of different powder samples were recorded using an XRD instrument (Malvern Panalytical, Almelo, the Netherlands). The scanning was performed over a 2θ range from 5° to 40°.

### 2.8 Fourier transform infrared spectroscopy (FTIR) analysis

The FTIR intensity of different samples was measured according to the method of (Tong et al., 2022) with slight modifications. FTIR spectra of the powder samples were recorded using a fourier transform infrared spectrometer (IR Affinity-1, Japan). A total of 64 scans were collected over a wavenumber range of 4000–500 cm^−1^ at an interval of 4 cm^−1^.

### 2.9 DPPH radical scavenging activity assay

The DPPH radical scavenging activity was measured based on the method of Yi et al. (2015) with minor modifications. A 2 mL aliquot of each diluted sample was mixed with 2 mL of 0.1 mM DPPH ethanol solution and incubated in the dark at room temperature for 30 min. A 50 % ethanol solution was used as the blank. The absorbance was measured at 517 nm. The radical scavenging activity was calculated as follows:

$$\text{Scavenging activity}\;\left(\%\right)=\left(1–\left(A_{s}-A_{b}\right)/A_{c}\right)\times 100$$

where A_s_ is the absorbance of the sample mixed with DPPH solution, A_b_ is the absorbance of the sample mixed with ethanol, and A_c_ is the absorbance of distilled water mixed with DPPH solution.

### 2.10 ABTS radical cation scavenging activity assay

The ABTS radical cation scavenging activity was determined according to the method of Yi et al. (2015) with slight modifications. ABTS stock solution (7.0 mM) was mixed with an equal volume of potassium persulfate solution (4.9 mM) and incubated in the dark for 12 h to prepare the ABTS^·+^ stock solution. The ABTS^·+^ working solution was prepared by diluting the stock solution with PBS buffer (10 mM, pH 7.0) to an absorbance of 0.70 ± 0.02 at 734 nm. The diluted sample solution was mixed with the ABTS^·+^ working solution at a ratio of 1:5 and reacted for 6 min. PBS was used as the blank. The absorbance was recorded at 734 nm, and the radical scavenging activity was calculated as follows:

$$\text{Scavenging activity}\;\left(\%\right)=\left(1–\left(A_{s}-A_{b}\right)/A_{c}\right)\times 100$$

where A_c_, A_s_, and A_b_ are the absorbance values of the control, sample, and sample without ABTS^·+^, respectively.

### 2.11 Ferric reducing antioxidant power (FRAP) assay

The FRAP assay was conducted following the method of Huang et al. (2025) with appropriate modifications. The FRAP reagent was prepared by mixing acetate buffer (300 mM, pH 3.6), ferric chloride solution (20 mM), and TPTZ solution (10 mM, dissolved in 40 mM HCl) in a 10:1:1 volume ratio. A 100 μL aliquot of each diluted sample was mixed with 3 mL of the FRAP reagent and incubated in the dark for 5 min. The absorbance was then measured at 593 nm. A standard curve was constructed using ascorbic acid, and the FRAP value was expressed as mg ascorbic acid equivalents per gram of dry weight (mg AAE/g DW).

### 2.12 Preparation of beef patties

Fresh beef tenderloin was trimmed to remove connective tissue and then minced in a blender (Nail, Zhejiang, China). An accurately weighed amount (50.0 ± 0.1 g) of the minced beef was mixed thoroughly with 0.5 g of WPI, WPIF, WR-NE, WR-E, WFR-NE, or WFR-E powder, and then molded into patties with the aid of a mold. The patties were baked in an o*v*en at 250 °C for 20 min (10 min per side). After cooling to room temperature, the baked patties were freeze-dried, ground, sieved, and stored at −20 °C until further analysis.

### 2.13 Determination of pH

Three grams of raw beef were minced and mixed with 25 mL of distilled water. The mixture was homogenized at 12,000 rpm for 1 min using a high-speed homogenizer (IKA T25, Digital Ultra-Turrax, Germany) and then allowed to stand at 4 °C for 30 min. The pH of the suspension was measured using a pH meter (FE20, Mettler-Toledo, Shanghai, China).

### 2.14 Determination of TBARS in roasted beef patties

TBARS values of the samples were determined based on the method of Zhang, Wang, et al. (2024) with modifications. Freeze-dried roasted beef meat samples (0.5 g each) were homogenized in 15 mL of 7.5 % (*w*/*v*) TCA solution at 8000 rpm for 1 min. After filtration, 2 mL of the filtrate was mixed with 2 mL of 20 mM TBA solution and heated at 95 °C for 30 min. After cooling to room temperature, absorbance was measured at 532 nm. A calibration curve based on 1,1,3,3-tetraethoxypropane was used to calculate the concentration of malondialdehyde (MDA).

### 2.15 Determination of rutin content in roasted beef patties

The extraction method for rutin was adopted with slight modifications from Chen, Lu, et al. (2024). Two grams of freeze-dried beef powder were mixed with 10 mL of 70 % ethanol and subjected to ultrasonic extraction at 40 °C for 30 min. The mixture was then centrifuged at 8000 rpm for 5 min, and the supernatant was collected. The extraction was repeated twice to ensure complete recovery of rutin, and the resulting supernatants were combined. The final extract was filtered through a 0.22-μm PTFE membrane and analyzed using UPLC-MS/MS on an ACQUITY UPLC H-Class system coupled with an Xevo TQ-XS triple quadrupole mass spectrometer equipped with an electrospray ionization (ESI) source (Waters Corp., USA). The chromatographic separation was carried out on a Waters ACQUITY UPLC BEH C18 column (1.7 μm, 2.1 × 100 mm). The mobile phase consisted of solvent A (acetonitrile) and solvent B (0.1 % formic acid aqueous solution), with a flow rate of 0.3 mL/min. The column temperature was set at 35 °C, the autosampler temperature at 10 °C, and the injection volume was 1 μL. The elution gradient was programmed as follows: 0–0.1 min, 10 % A; 0.1–4 min, 10–100 % A; 4–5 min, 100–10 % A; and 5–6 min, 10 % A. Mass spectrometry was performed using an electrospray ionization (ESI) source in negative ion mode with the following settings: capillary voltage, 3 kV; desolvation temperature, 550 °C; desolvation gas flow, 1000 L/h; collision gas flow, 0.16 mL/min; and source temperature, 150 °C.

### 2.16 Extraction and determination of HAs

Accurately weighed 1 g of freeze-dried meat powder was placed into a 50 mL centrifuge tube, and 10 mL of 1 mol/L NaOH solution was added. The mixture was vortexed for 5 min and then mixed with 5 g of diatomaceous earth, followed by ultrasonication for 30 min. Subsequently, 20 mL of ethyl acetate was added and sonicated for an additional 30 min. The mixture was centrifuged at 8000 rpm for 5 min to collect the supernatant. The extraction procedure was repeated twice to ensure complete extraction of free HAs, and the ethyl acetate extracts were combined. The pooled extract was evaporated under nitrogen at 50 °C to a final volume of approximately 20 mL. Then, 100 μL of 2 mol/L HCl was added to neutralize the residual NaOH. The resulting solution was subjected to solid-phase extraction using an Oasis MCX cartridge. The eluate was evaporated to dryness under nitrogen and reconstituted in 300 μL of methanol. After filtration through a 0.22 μm membrane, the sample was analyzed by UPLC-MS/MS. The detailed procedure followed the method of  Chen, Yi, et al. (2024).

### 2.17 Extraction and determination of CML and CEL

A total of 50 mg of freeze-dried meat powder was mixed with 2 mL of borate buffer (0.2 M, pH 9.2) and 0.4 mL of sodium borohydride solution (2 M in 0.1 M NaOH). After releasing the generated gas bubbles, the mixture was transferred to a 4 °C refrigerator and incubated overnight for reduction. After reduction, 2.4 mL of concentrated hydrochloric acid was added to adjust the final HCl concentration to 6 M. The mixture was hydrolyzed at 110 °C for 24 h. The hydrolysate was filtered and diluted to a final volume of 10 mL with ultrapure water. Then, 1 mL of the hydrolysate was evaporated to dryness under nitrogen at 50 °C. The residue was reconstituted with 2 mL of ultrapure water, and 100 μL of internal standards d₄-CML and d₄-CEL (0.5 μg/mL) were added. The mixture was purified using an Oasis MCX solid-phase extraction cartridge. The eluate was evaporated under nitrogen and reconstituted in 500 μL of ultrapure water. After filtration through a 0.22 μm membrane, the sample was subjected to LC-MS/MS analysis. The procedure was based on the method described by Chen, Lu, et al. (2024).

### 2.18 Determination of amino acid content

Freeze-dried roasted beef samples (1 g each) were mixed with 25 mL of 5 % TCA solution and ultrasonically extracted for 1 h. The mixture was filtered through a 0.22 μm syringe filter. Chromatographic conditions: Waters ACQUITY HSS T3 column (150 × 2.1 mm, 1.7 μm); mobile phases: A (0.1 % formic acid aqueous solution) and B (0.1 % formic acid acetonitrile solution); flow rate: 0.2 mL/min; column temperature: 35 °C; sample chamber temperature: 10 °C; injection volume: 2 μL. The gradient elution program was as follows: 0–3 min, 100 % A; 3–6 min, 100 %–90 % A; 6–10 min, 90 %–65 % A; 10–11 min, 65 %–5 % A; 11–13.5 min, 5 % A; 13.5–18 min, 5 %–95 % A. Mass spectrometry conditions employed the ESI positive ion mode, and all other conditions were the same as those described in 2.15.

### 2.19 Statistical analysis

All quantitative experiments were carried out in triplicate and the results are reported as the arithmetic mean ± standard deviation (SD). The data were statistically analyzed using SPSS Statistics 20 with one-way analysis of variance (ANOVA), followed by LSD and Duncan's multiple range tests, with a confidence level of 0.05. Principal component analysis (PCA) was performed using SIMCA 14.1. All figures and charts were created using Origin 2025, and standard curve construction and compound content determinations were conducted with Excel 2021.

## 3 Results and discussion

### 3.1 Characterization of WPI and WPIF complexes

#### 3.1.1 ThT fluorescence analysis

**Fig. 1:**
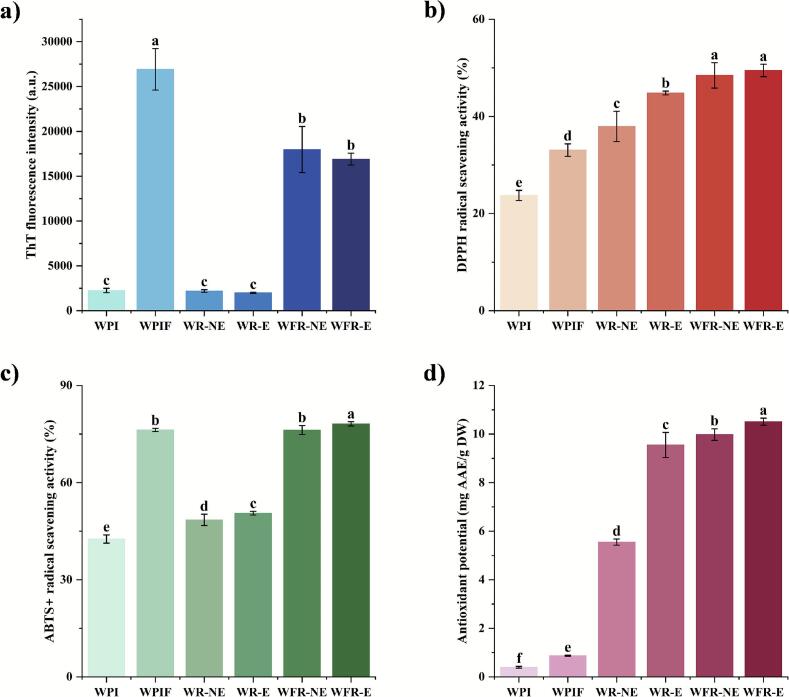
ThT fluorescence intensity (a), DPPH radical scavenging activity (b), ABTS radical cation scavenging activity (c), and FRAP (d) of WPI, WPIF, and their complexes with rutin. Values are expressed as mean ± standard deviation (SD) (*n* = 3). Lowercase letters (a-f) indicate significant differences among different samples (*P* < 0.05).

ThT fluorescence assay has been widely used for detecting fibrils due to the high binding affinity of ThT with β-sheet-rich structures (Chen, Yi, et al., 2024). As shown in Fig. 1a, WPI exhibited weak fluorescence, suggesting a low content of β-sheet structures. In contrast, its conversion to fibrils resulted in substantially enhanced (>10-fold) fluorescence intensity, indicating a much higher β-sheet content and confirming the successful formation of WPIF. The data also showed that the presence of rutin in the WPIF-based samples, regardless of encapsulated or free form, led to significantly attenuated fluorescence intensity. This observation was consistent with previous studies which found that hydrophobic interactions between polyphenols and legume proteins significantly influenced β-sheet formation with a general trend toward reduction in β-sheet content of the protein fibrils and thus ThT fluorescence intensity (Tong et al., 2022).

#### 3.1.2 Antioxidant capacity analysis

The antioxidant properties of WPI, WPIF, and their rutin complexes are shown in Fig. 1b-d. Compared to WPI, the WPIF samples exhibited markedly enhanced (*P* < 0.05) antioxidant capacity by 9.35 % in the DPPH radical scavenging, 33.70 % in the ABTS radical cation scavenging, and 0.47 mg AAE/g DW in the FRAP assay. This enhancement might be attributed to the structural changes which caused exposure of more functional amino acid residues in the acid-heat-induced fibril formation process. In particular, the exposure of aromatic and sulfur-containing amino acid residues could enhance its antioxidant properties (Feng et al., 2022; Mohammadian & Madadlou, 2016). Moreover, the encapsulation of rutin in the WR-E group further improved its antioxidant performance compared to the WR-NE group whose rutin was in a non-encapsulated state. Specifically, the DPPH, ABTS, and FRAP values of the WR-E group was 6.88 %, 2.05 %, and 4.00 mg AAE/g DW higher than those of the WR-NE group, respectively (*P* < 0.05). Significant differences were also observed between WFR-E and WFR-NE. These findings support that the incorporation/encapsulation of rutin could effectively preserve its antioxidant property likely through protein–polyphenol interactions (Guo et al., 2023). It was also noted that the WPIF-rutin complexes had higher antioxidant activity than the WPI-rutin complexes in the DPPH (4.64 %), ABTS (27.60 %), and FRAP (0.96 mg AAE/g DW) assay, respectively, which may be attributed to two main mechanisms. First, WPIF likely formed more stable complexes with rutin than did WPI that eventually facilitated its effective interaction and thus scavenging of free radicals in the system (Ji et al., 2023). The former complex might also confer better protection of rutin's chemical properties, consistent with Hu et al.'s report of the effectiveness of fibrillar protein in protecting the hydroxyl functional groups of polyphenols (Hu et al., 2020). Second, binding of rutin with WPIF might result in more favorable structural alteration than that with WPI in the protein matrix, leading to exposure of more functional groups with antioxidant potential (Li et al., 2024; Singh et al., 2011).

#### 3.1.3 SDS-PAGE analysis

**Fig. 2:**
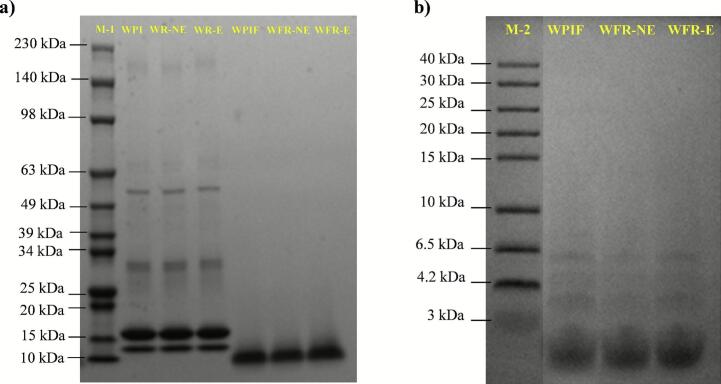
(a) SDS-PAGE profiles of WPI, WPIF, and their complexes with rutin. M-1: molecular weight marker (10–230 kDa); (b) SDS-PAGE profiles of WPIF and its complexes with rutin. M-2: molecular weight marker (3–40 kDa).

The SDS-PAGE results of different samples are shown in Fig. 2. In WPI, clear bands were observed at 14 and 18 kDa, corresponding to α-lactalbumin and β-lactoglobulin, respectively, along with higher molecular weight bands for bovine serum albumin (65 kDa) and immunoglobulin (150 kDa) (Meng et al., 2024). In WPIF, all bands observed in WPI, including α- and β-lactoglobulin, disappeared, and new bands appeared around 10 kDa, likely corresponding to peptide fragments generated during the acid-heat treatment, which may contribute to fibril formation (Zhang et al., 2021). Further analysis with low-molecular-weight Tricine-SDS-PAGE confirmed the presence of peptide bands in the 3–6.5 kDa and < 3 kDa range in WPIF. The generation of <6.5 kDa peptides after acid-heat treatment of WPI was also reported by Zhang et al. (2021). These peptides have been demonstrated to possess enhanced antioxidant activity due to better solubility, higher flexibility, and more active amino acid residues (Ballatore et al., 2020; Yaghoubzadeh et al., 2020). These findings are consistent with the superior antioxidant capacity of WPIF presented in section 3.1.2. Notably, the addition of rutin did not alter the protein band distribution in the WPI and WPIF samples.

#### 3.1.4 TG analysis

**Fig. 3:**
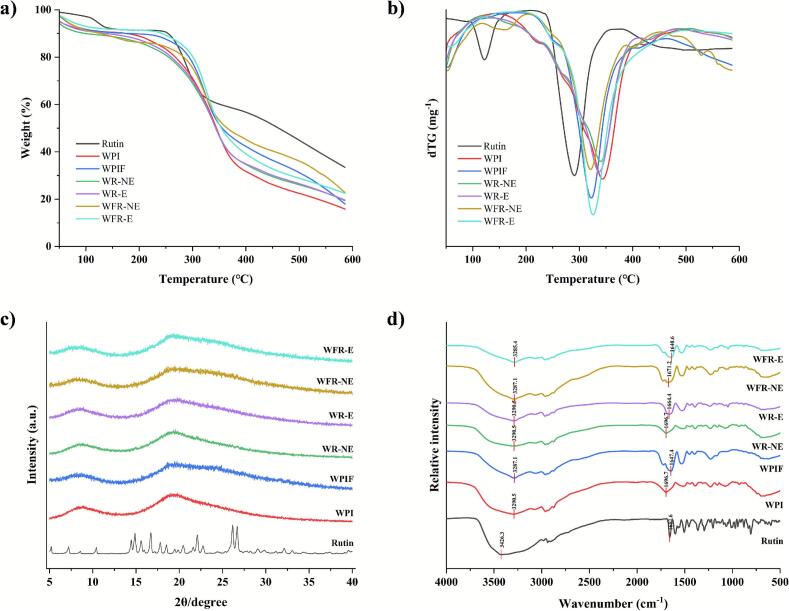
TG (a), dTG (b), XRD (c), and FTIR spectra (d) of rutin, WPI, WPIF, and their complexes with rutin.

TG analysis is widely employed to evaluate mass changes and thermal stability during heating. Typically, mass loss arises from solvent evaporation and thermal degradation (Ricci et al., 2018). As shown in Fig. 3a, pure rutin exhibited minimal mass losses at low temperatures, indicating a low moisture content. A major decomposition peak was observed at 290 °C, with 66.57 % mass loss at 600 °C. Appreciable weight losses were noted in WPI and WPIF at around 100 °C due to moisture evaporation and greater losses occurred between 320 and 350 °C likely due to more significant structural degradation. Residual weights at 600 °C for WR-NE, WR-E, WFR-NE, and WFR-E were 19.61 %, 19.34 %, 22.77 %, and 22.50 %, respectively. Mass losses of the WPIF-rutin complexes were smaller than those of the WPI-rutin complexes, indicating higher thermal stability probably due to stronger molecular interactions in the former samples (Hu et al., 2021). Meng et al. (2024) also found higher residual levels of curcumin and piperine with WPIF than with WPI encapsulation. At 250 °C (representing thermal condition for preparation of roast beef patties), WR-NE, WR-E, WFR-NE, and WFR-E retained 80.84 %, 81.85 %, 84.39 %, and 89.31 % of their weight, respectively, consistent with the trends at 600 °C.

#### 3.1.5 XRD and FTIR analysis

XRD was employed to determine crystalline structures of different powders. As shown in Fig. 3c, rutin exhibited multiple sharp peaks at 14.89°, 15.59°, 22.10°, 26.16°, and 26.71°, indicating its crystalline nature. In contrast, WPI and WPIF showed broad humps at around 8.0–9.3° and 18.5–20.3°, indicating amorphous structures, consistent with previous reports (Ye et al., 2018). None of the complex samples (WR-NE, WR-E, WFR-NE, WFR-E) displayed characteristic peaks of rutin, suggesting that rutin was likely in an amorphous state and/or successfully embedded/encapsulated in the protein. Similar results were also reported in protein-polyphenol complexes, such as ovalbumin amyloid fibrils with resveratrol (Wang et al., 2024). Interestingly, the absence of characteristic sharp peaks also happened in those samples where rutin was merely mixed with the respective protein physically (WR-NE and WFR-NE). This latter observation suggested that the high protein-to-rutin ratio (50:1) may have masked the rutin signal. Further research is needed to elucidate this phenomenon.

Fig. 3d presents the FTIR spectra of different samples. Significant changes were observed in the amide A band (3200–3400 cm^−1^) associated with O—H and N—H stretching vibrations, and the amide I band (1700–1600 cm^−1^) corresponding to C=O stretching vibrations (Tong et al., 2022). Compared with WPI, the amide A absorption peak of WPIF shifted from 3290.5 cm^−1^ to 3287.1 cm^−1^, indicating enhanced hydrogen bonding during fibril formation (Zhao et al., 2025). The addition of rutin did not cause any significant changes in the positions of the absorption peaks (WR-NE and WR-E versus WPI), and the pattern of WFR-NE was also largely consistent with that of WPIF. Meanwhile, compared to WPIF, further shifting of the amide A band of WFR-E to 3285.4 cm^−1^ suggested the formation of hydrogen bonds between rutin and the protein fibrils (Tong et al., 2022). The shift of the amide I band from 1696.7 cm^−1^ in WPI to 1647.4 cm^−1^ in WPIF may be attributed to changes in the secondary structure of the protein, particularly random coil to β-sheet transition (Zhou et al., 2022). This observation is consistent with the ThT fluorescence data shown in Fig. 1a. Further red shifts of the amide I absorption peaks in the rutin-encapsulated samples WR-E and WFR-E to 1664.4 cm^−1^ and 1640.6 cm^−1^ respectively might be due to hydrophobic interactions between rutin and the C=O groups of the protein (Zhao et al., 2020).

### 3.2 Effects of WPI and WPIF with or without rutin on the quality of roasted beef patties

#### 3.2.1 Analysis of pH, TBARS, and thermal degradation rate of rutin

**Fig. 4:**
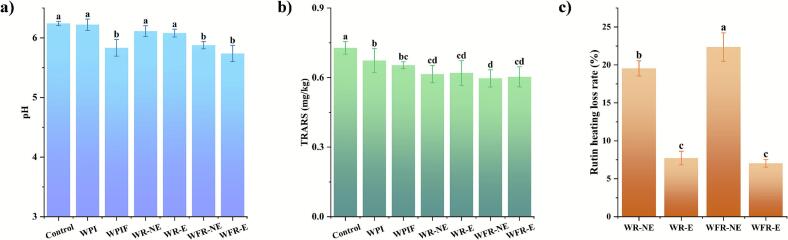
pH values of raw beef in different samples (a), TBARS values of roasted beef patties (b), and thermal degradation rate of rutin (c). Values are expressed as mean ± standard deviation (SD) (n = 3). Lowercase letters (a-d) indicate significant differences among different samples (*P* < 0.05).

The effects of WPI, WPIF, and their rutin complexes on the pH of raw beef patties, TBARS values of roasted beef patties, and thermal degradation rate of rutin are presented in Fig. 4. As shown in Fig. 4a, the pH values of the WPIF- and its rutin complex-pretreated groups were significantly lower than those of the control and WPI-pretreated groups (*P* < 0.05). In particular, the pH values of the WPIF, WFR-NE, and WFR-E groups decreased by 6.52 %, 5.82 %, and 8.07 %, respectively, compared to the control. The decrease in pH could be ascribed to the acidic conditions (pH = 2) used for the preparation of WPIF. This pH-lowering effect upon addition of the WPIF complexes to the beef patties may therefore not only inhibit microbial growth but also slow down the Maillard reaction during thermal treatment (El Hosry et al., 2025).

In the TBARS assay to evaluate the relative levels of lipid oxidation in the roasted beef patties (Fig. 4b), both the WPI and WPIF groups were found to have significantly lower contents of TBARS than those of the control (*P* < 0.05). No significant difference existed between WPI and WPIF, suggesting that the enhancement in the antioxidant activity due to the structural modification observed in the in vitro antioxidant assays (section 3.1.2) was not effectively translated into inhibition of lipid oxidation in complex meat systems. Further analysis showed that all the rutin-incorporated groups (WR-NE, WR-E, WFR-NE, and WFR-E) had significantly lower TBARS levels than the WPI group (*P* < 0.05), consistent with its intrinsic antioxidant capacity and indicative of its effective preservation in these complexes to subsequently exert its protective effect against heat-induced lipid oxidation in the beef patties (Soendjaja & Girard, 2024). Among them, the TBARS values for WFR-NE and WFR-E were 0.597 ± 0.037 and 0.603 ± 0.044, respectively, slightly lower than WR-NE (0.615 ± 0.037) and WR-E (0.620 ± 0.053), though the differences were not significant.

Fig. 4c presents the thermal degradation rate of rutin during the roasting process of the beef patties. Rutin loss was 19.55 % and 22.36 % in the WR-NE and WFR-NE group, respectively, while it was only 7.73 % and 7.03 % in the WR-E and WFR-E (*P* < 0.05) group, indicating that both the WPI and WPIF encapsulation systems significantly improved rutin thermal stability. Notably, WFR-E demonstrated the most effective protection for rutin in the beef patties, and this was consistent with the data in the thermogravimetric analysis at 600 °C, which showed a higher residual level of rutin than the other encapsulation systems tested. Previous studies also reported the superior protective effect of WPIF on polyphenols under high-temperature conditions (Farrokhi et al., 2019).

#### 3.2.2 Changes in HAs content in roasted beef patties

The effects of WPI, WPIF, and their rutin complexes on HAs content in roasted beef patties are shown in Table 1. The total HAs content in the control group was 31.84 ± 1.89 ng/g. WPI alone promoted the formation of HAs by 7.54 %, especially PhIP, and MeIQx. This might be due to the addition of Maillard-active amino acids to the meat matrix from heat-induced partial hydrolysis of WPI that eventually enhanced reactions associated with HA formation (Li et al., 2023). Conversely, the WPIF group exhibited a reduction in total HAs to 27.00 ± 0.70 ng/g (a 15.20 % decrease), consistent with its strong antioxidant activity observed in section 3.2. A closer examination of the individual HAs revealed that both WPI and WPIF had a moderate promoting effect on the formation of quinoxaline-type HAs, including 4,8-DiMeIQx, 7,8-DiMeIQx, and MeIQx. This phenomenon will be further discussed in section 3.2.4.

**Table 1:** Effects of WPI, WPIF, and their complexes with rutin on heterocyclic amine contents in roasted beef patties (ng/g).

	Control	WPI	WPIF	WR-NE	WR-E	WFR-NE	WFR-E
IQx	0.14 ± 0.02^a^	0.14 ± 0.02^a^	0.11 ± 0.01^b^	0.14 ± 0.02^a^	0.09 ± 0.01^c^	0.11 ± 0.01^bc^	0.09 ± 0.01^c^
MeAaC	0.44 ± 0.06^a^	0.47 ± 0.04^a^	0.35 ± 0.04^b^	0.30 ± 0.02^c^	0.27 ± 0.03^c^	0.27 ± 0.02^c^	0.21 ± 0.01^d^
AaC	6.18 ± 0.57^ab^	6.25 ± 0.27^ab^	5.24 ± 0.22^de^	5.75 ± 0.42^bc^	5.44 ± 0.27^cd^	4.88 ± 0.08^ef^	4.44 ± 0.21^f^
Harmane	14.37 ± 1.16^a^	14.90 ± 0.49^a^	10.24 ± 0.50^b^	8.86 ± 0.37^c^	8.69 ± 0.79^c^	8.01 ± 0.18^cd^	7.56 ± 0.29^d^
Norharmane	0.16 ± 0.02^b^	0.15 ± 0.01^bc^	0.19 ± 0.01^a^	0.13 ± 0.01^c^	0.13 ± 0.01^c^	0.14 ± 0.01^bc^	0.13 ± 0.01^c^
PhIP	3.75 ± 0.11^b^	4.58 ± 0.22^a^	3.05 ± 0.19^c^	2.89 ± 0.03^c^	2.97 ± 0.04^c^	2.68 ± 0.13^d^	2.37 ± 0.11^e^
4,8-DiMeIQx	2.96 ± 0.17^bc^	3.12 ± 0.07^b^	3.41 ± 0.23^a^	2.83 ± 0.11^cd^	2.71 ± 0.05^d^	2.81 ± 0.05^cd^	2.70 ± 0.18^d^
7,8-DiMeIQx	1.98 ± 0.08^b^	2.56 ± 0.22^a^	2.15 ± 0.10^b^	1.26 ± 0.17^c^	1.24 ± 0.07^c^	0.85 ± 0.04^d^	0.89 ± 0.02^d^
MeIQx	1.86 ± 0.10^bc^	2.06 ± 0.09^ab^	2.24 ± 0.21^a^	1.77 ± 0.12^cd^	1.73 ± 0.19^cd^	1.86 ± 0.18^bc^	1.61 ± 0.13^d^
Total HAs	31.84 ± 1.89^b^	34.24 ± 0.31^a^	27.00 ± 0.70^c^	23.92 ± 0.66^d^	23.26 ± 0.92^d^	21.61 ± 0.25^e^	20.00 ± 0.44^f^

Lowercase letters (a-f) indicate significant differences in the content of the same heterocyclic amine among different samples (*P* < 0.05), *n* = 3.

The rutin-containing groups, WR-NE, WR-E, WFR-NE, and WFR-E, all showed significantly lower total HAs contents than the control by 24.87 %, 26.93 %, 32.12 %, and 37.18 %, respectively. This reinforced the HA-inhibitory effectiveness of rutin that may be mediated through the scavenging or trapping of radicals and reactive carbonyls (Zhang et al., 2023). Moreover, WFR-E showed the strongest inhibition with regard to MeAaC, PhIP, MeIQx, and total HAs, likely due to the stabilization and thus better preservation of rutin's functional moieties, especially hydroxyl groups, in the fibril-structured complex (Farrokhi et al., 2019; Hu et al., 2020).

#### 3.2.3 Changes in CML and CEL content in roasted beef patties

As shown in Table 2, WPI, WPIF, and their rutin complexes significantly affected CML and CEL levels in roasted beef patties. The CML and CEL contents of the control group were 4.88 ± 0.61 μg/g and 11.53 ± 0.85 μg/g, respectively. WPI increased these levels by 14.73 % and 7.55 %, likely due to the addition of proteins and subsequently more substrates for glycation reactions to produce CML and CEL (Prestes Fallavena et al., 2022). In contrast, the fibrillar form WPIF, reduced CML, CEL, and total AGEs by 19.80 %, 19.03 %, and 19.26 %, respectively, compared to the control, which was likely brought about by its superior antioxidant capacity.

**Table 2:** Effects of WPI, WPIF, and their complexes with rutin on the contents of CML, CEL, and total AGEs in roasted beef patties (μg/g).

	CML	CEL	Total AGEs
Control	4.88 ± 0.61^a^	11.53 ± 0.85^b^	16.41 ± 0.99^b^
WPI	5.59 ± 0.31^b^	12.40 ± 0.46^a^	17.99 ± 0.28^a^
WPIF	3.91 ± 0.34^c^	9.34 ± 0.32^cd^	13.25 ± 0.57^cd^
WR-NE	3.62 ± 0.50^cd^	9.91 ± 0.31^c^	13.53 ± 0.49^c^
WR-E	3.49 ± 0.44^cde^	9.43 ± 0.38^cd^	12.92 ± 0.66^cd^
WFR-NE	3.27 ± 0.11^de^	9.24 ± 0.25^cd^	12.51 ± 0.30^d^
WFR-E	2.93 ± 0.14^e^	8.73 ± 0.45^d^	11.66 ± 0.31^e^

Lowercase letters (a-e) indicate significant differences in the content of the same AGEs among different samples (*P* < 0.05), n = 3.

In both the WPI- and the WPIF-based systems, the presence of rutin led to significant reductions in the contents of CML, CEL, and total AGEs compared with the control. It was noted that the treatments containing rutin in the encapsulated form consistently performed better than the ones containing it without encapsulation. For instance, WR-E reduced AGEs content by 21.3 % while WR-NE reduced it by 17.5 % relative to the control. These values also represented a 28.2 % and 24.8 % reduction compared to that of the WPI group, highlighting that the inhibitory effect of rutin on AGEs formation was probably owing to its carbonyl trapping and radical scavenging activity (Chen et al., 2022). Moreover, the total AGEs contents of the WFR-NE and WFR-E groups were 12.51 ± 0.30 and 11.66 ± 0.31 μg/g, representing a further reduction of 7.5 % and 9.8 % compared to WR-NE and WR-E, respectively (*P* < 0.05). These data therefore support that the encapsulation of rutin in WPIF could effectively preserve its antioxidant capacity, and represents an effective approach to realize their combined inhibitory activity against AGE formation in roasted beef (Farrokhi et al., 2019).

#### 3.2.4 Changes in amino acid contents of roasted beef patties subjected to different treatments

As discussed in section 3.2.2, the moderate promoting effect of WPI and WPIF on the formation of quinoxaline HAs might be partly brought about by the release of Maillard-active amino acids to the meat matrix from heat-induced hydrolysis of the proteins. To verify this proposition, the amino acid profiles of the roasted beef patties from the various groups were determined (Table 3). The results showed that multiple amino acids, especially glycine (Gly) and threonine (Thr) were significantly elevated in the beef patties pretreated with the WPIF-based test agents. Gly content in WPIF reached 899.87 nmol/L (4.99-fold higher than WPI), and Thr was 436.55 nmol/L (44.32-fold higher). Glycine and threonine (Thr) have been demonstrated to be among the major precursor amino acids for the formation of quinoxaline-type HAs (Caro-Cabarcas et al., 2025). These data may partly explain the enhanced quinoxaline HAs formation in the WPIF-added roasted beef patties without any additional antioxidant partner (rutin). Literature data together with ours also emphasize the importance of amino acid profile changes when investigating the intervention mechanisms of exogenous agents on the formation of HAs in meat products (Geng et al., 2024).

**Table 3:** Differences in amino acid concentrations among WPI, WPIF, and their rutin complexes (nmol/L).

	Gly	Thr	Phe	Trp	Arg	Lys
WPI	150.33 ± 14.93^c^	9.63 ± 0.93^c^	8.00 ± 0.66^d^	2.73 ± 0.15^d^	0.82 ± 0.08^a^	1.43 ± 0.06^c^
WPIF	899.87 ± 27.93^a^	436.55 ± 47.92^a^	79.65 ± 6.80^a^	24.32 ± 2.06^a^	0.77 ± 0.06^ab^	9.57 ± 0.06^ab^
WR-NE	69.30 ± 7.36^d^	6.40 ± 0.46^c^	5.33 ± 0.12^d^	2.13 ± 0.12^d^	0.75 ± 0.05^ab^	1.00 ± 0.10^c^
WR-E	64.61 ± 1.70^d^	5.00 ± 0.03^c^	5.04 ± 0.07^d^	2.10 ± 0.23^d^	0.69 ± 0.02^b^	0.99 ± 0.03^c^
WFR-NE	825.63 ± 21.50^b^	342.73 ± 6.40^b^	71.42 ± 1.32^b^	16.44 ± 1.32^b^	0.71 ± 0.06^b^	9.90 ± 0.30^a^
WFR-E	845.32 ± 47.34^b^	325.64 ± 18.11^b^	66.08 ± 1.57^c^	12.97 ± 1.66^c^	0.67 ± 0.02^b^	9.20 ± 0.62^b^

Lowercase letters (a-d) indicate significant differences in the content of the same amino acid among different samples (*P* < 0.05), n = 3.

Although the levels of phenylalanine (Phe, a precursor of PhIP (Deng, Xue, et al., 2022)) and tryptophan (Trp, a precursor of harmane and norharmane (Xi & Chen, 2021)) increased by 8.96- and 7.90-fold, respectively, the corresponding HAs contents decreased, suggesting that the antioxidant capacity of WPIF was sufficient to suppress their formation pathways. The acid-heat treatment process for the transformation of WPI to WPIF might cause the exposure of amino acid residues with thiol or aromatic side chains that subsequently contributed to the enhanced antioxidant potential, thereby disrupting free radical- and reactive carbonyl-mediated reactions (Zhao et al., 2021). Similarly, the much higher level of lysine (Lys), an important AGEs precursor, in the WPIF than the WPI group did not concur with their relative modulating effect on the AGEs contents of the roasted beef patties.

The above findings supported the potential of WPIF both as an improved derivative of WPI and as a promising carrier for rutin to realize their combined favorable modulating effect on the contents of hazardous heat-induced by-products in meat products.

### 3.3 Principal component analysis

**Fig. 5:**
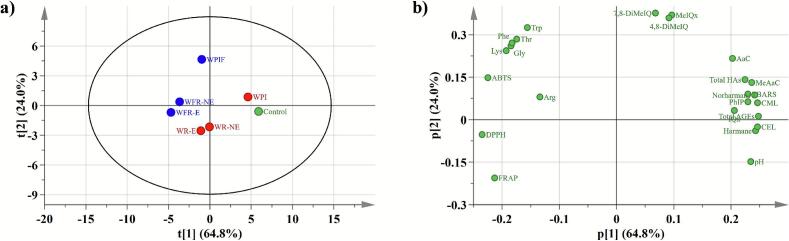
Principal component analysis of the physicochemical properties of WPI, WPIF, and their complexes with rutin, and their effects on the quality and safety of beef patties: (a) score plot and (b) loading plot.

To better visualize the effects of WPI, WPIF, and their complexes on the quality of roasted beef patties, principal component analysis (PCA) was conducted on key indices assayed. Fig. 5 presents the PCA score plot (a) and loading plot (b), with PC1 and PC2 cumulatively explaining 88.8 % of the variance, sufficiently capturing inter-variable relationships. The sample groups were distinctly separated along PC1, indicating that the different pretreatments significantly influenced physicochemical properties of the roasted beef patties. The control and WPI groups were located in the positive PC1 direction, corresponding with their higher levels of lipid oxidation, HAs, and AGEs. Conversely, the WFR-E group in the negative PC1 direction corresponded with their superior performance in inhibition of oxidation and harmful byproduct formation. Fig. 5b shows that the contents of TBARS, CML, CEL, and most non-quinoxaline HAs were positively correlated and clustered in the positive PC1 direction, aligning with the control and WPI positions. WPIF aligned with quinoxaline HAs (MeIQx, 4,8-DiMeIQx, 7,8-DiMeIQx) and many amino acids, concurring with the results in 3.2.2, 3.2.3. The antioxidant indicators (DPPH, ABTS, FRAP) were located in the negative PC1 quadrants, reflecting the high antioxidant capacity of the WPIF-rutin complexes. In summary, the PCA results have further confirmed the significant influence of WPI, WPIF, and their rutin complexes on the antioxidant properties and formation of HAs and AGEs in roasted beef patties.

## 4 Conclusion

This study systematically investigated the physicochemical properties of WPI, WPIF, and their combinations with rutin through simple physical mixing and in an encapsulated form, as well as their effects on lipid oxidation, HAs, and AGEs formation in roasted beef patties. WPIF was found to possess significantly higher antioxidant activity compared to WPI in the DPPH (39.41 %), ABTS (79.14 %), and FRAP (0.47 mg AAE/g DW) assays. This was likely due to the low-molecular-weight peptides that were derived from the acid-heat treatment in the conversion of WPI to WPIF. Encapsulation of rutin through hydrogen bonding and hydrophobic interactions resulted in a complex (WFR-E) with improved properties, especially thermal stability and antioxidant capacity. The positive attributes were also demonstrated in roasted beef patties, where the thermal degradation rate of rutin in the encapsulated form (WR-E and WFR-E) was lower than that of the non-encapsulated form (∼7 % vs. ∼20 %). Subsequently, adverse heat-induced reactions, particularly lipid peroxidation (TBARS), HAs, and AGEs formation, in the WFR-*E*-pretreated roasted beef patties were significantly attenuated compared to those in the control. Although WPIF addition led to higher contents of precursors for quinoxaline-type HAs (e.g., Gly and Thr), encapsulation with rutin effectively mitigated this potential negative impact as evidenced by the significant inhibition of 4,8-DiMeIQx, 7,8-DiMeIQx, and MeIQx formation in the WFR-E group relative to the control. Notably, WFR-E-pretreated roasted beef patties had the lowest contents of total HAs and AGEs among all the study groups. Although polphenols have been widely demonstrated to be among the most promising classes of natural agents for modulation of a range of quality attributes in food products, the poor water solubility and thermal stability have remained a major shortcoming limiting the broad application of many candidates. The findings from the present study support that encapsulation of rutin and other polyphenols with similar chemical properties in WPIF may be an effective strategy to overcome the limitation, and thus facilitate their application in thermally processed meat products to enhance their quality and safety.

## CRediT authorship contribution statement

**Zening Zhang:** Writing – review & editing, Writing – original draft, Methodology, Data curation, Conceptualization. **Qiaochun Chen:** Writing – review & editing, Methodology. **Muhammad Aslam Khan:** Writing – review & editing, Methodology. **Baoping Shi:** Writing – review & editing. **Huaixu Wang:** Writing – review & editing. **Ka-Wing Cheng:** Writing – review & editing, Supervision, Resources, Project administration, Methodology, Funding acquisition, Conceptualization.

## Declaration of competing interest

The authors declare that they have no known competing financial interests or personal relationships that could have appeared to influence the work reported in this paper.

## Data Availability

Data will be made available on request.
